# Hydatidosis of the complete humerus. Treated with radical resection and endoprosthesis. Case report

**DOI:** 10.1016/j.ijscr.2019.10.083

**Published:** 2019-11-04

**Authors:** Juan Martin Patino, Alejandro José Ramos Vertiz

**Affiliations:** Departamento de Ortopedia y Traumatologia, Servicio de cirugía de mano y miembro superior, Hospital Militar Central “Cosme Argerich”, Buenos Aires, Argentina

**Keywords:** Hydatidosis, Total humerus, Humerus endoprosthesis, Case report, Limb salvatage

## Abstract

**Introduction:**

Hydatid bone disease is caused by the *Echinococcus granulosus worm.* The location of the disease in the humerus is infrequent.

No reports of hydatid disease in the entire humerus have been found in the bibliography. We present one case of primary hydatid bone disease affecting the entire humerus, which was treated with radical resection and total endoprosthesis of the humerus.

**Case:**

A 24-year-old patient presented with a diaphyseal humerus fracture. The x-rays and MRI showed an oblique fracture and heterogeneous osteolytic and multiloculated images along the entire humerus. The biopsy resulted in hydatid cysts. Oncological resection of the humerus and total replacement of the same with a non-conventional prosthesis designed for the patient was performed.

**Conclusion:**

Total humerus hydatidosis is infrequent, and there is scarce information about its treatment. The local resection plus graft and osteosynthesis conserving the bone has shown complications and recurrences. For healing, a radical procedure is necessary. Although functional results may be limited.

## Highlights

• Hydatid bone disease is caused by the *Echinococcus granulosus* worm. Osseous involvement accounts for 0.5% to 4% of cases in humans.
• No reports of hydatid disease in the entire humerus have been found in the bibliography.
• There is no consensus as regards the medical treatment of hydatidosis in the humerus.
• The affections of the entire humerus and the recurrences present a challenge of treatment for the reconstruction and rescue of the limb.
• We report a case of salvatage limb with a total humerus endophrostesis with 2 years of follow up.

## 1 Introduction

Hydatid bone disease is caused by the *Echinococcus granulosus worm*. This zoonosis is endemic in the Mediterranean, Middle East, Asia, Africa and many South American countries, and it is a major public health and economic problem in the Patagonian region of Argentina. Osseous involvement accounts for 0.5 %–4 % of cases in humans. The spine (35 %) and the pelvis (21 %) are the most commonly involved skeletal sites, followed by the long bones, especially the femur (16 %) [1]. The location of the disease in the humerus is infrequent. Epidemiological research in some regions has shown: 3 cases of hydatidosis of the humerus between 1971 and 2010 in Serbia, 1 case between 2010 and 2017 in Kazagistan, and no cases have been reported in Spain between 1989 and 2017 [1]. Out of 329 cases of hydatidosis reported between 2005 and 2016, only one case of hydatidosis in the humerus was reported in Turkey [2].

There is no consensus as regards the medical treatment of hydatidosis in the humerus. Local procedures such as partial resection, curettage, and bone grafting or filling with cement PMMC POLIMETILMETACRILATO were indicated. More severe or extended cases, or where complications such as pathological fractures have occurred, require mega prosthesis or massive allograft for reconstruction [3,4]. Another radical option is the limb ablation, but the rescue surgery often has better results and is more easily tolerated by the patient, than complete limb amputation, both emotionally and socially [5,6].

The use of allografts, alloprosthetic composites and prosthetic replacements to reconstruct the proximal humerus after tumor resection and associated complications, have been well described [7]. However, the affections of the entire humerus and the recurrences present a challenge of treatment for the reconstruction and rescue of the limb [8].

No reports of hydatid disease in the entire humerus have been found in the bibliography. This research presents one case of primary hydatid bone disease affecting the entire humerus, which was treated with radical resection and total endoprosthesis of the humerus.

This work has been reported in line with the SCARE criteria [9], and it has been performed in a private academic hospital.

**Fig. 1:**
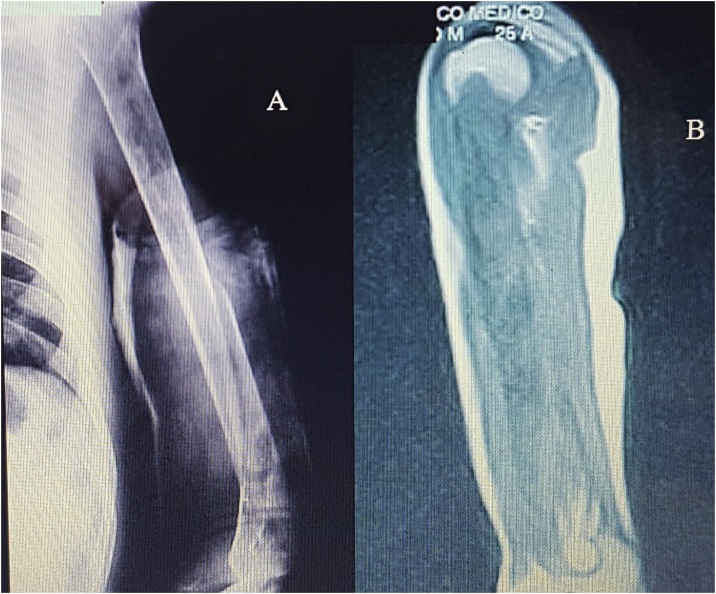
An AP x-rays showing humeral shaft fracture. B MRI showing total humerus compromise.

Informed consent was obtained from the patient included in the study, and the institutional Ethical Committee approved the retrospective medical chart review Fig. 1.

**Fig. 2:**
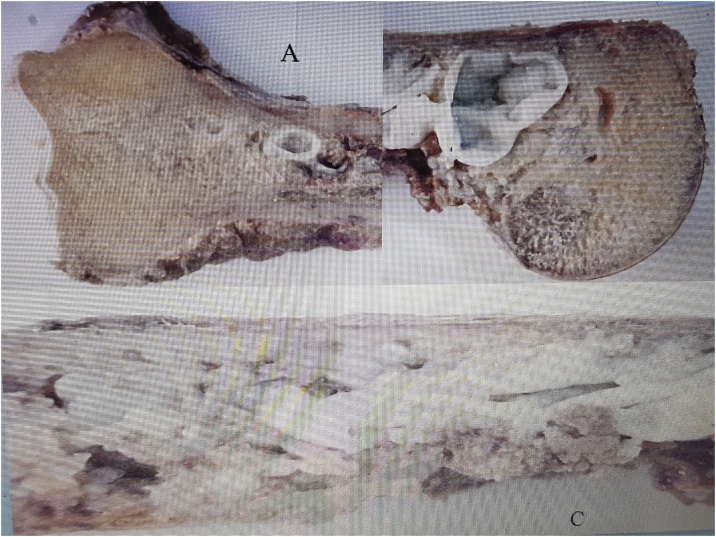
A–C macroscopic humeral image with proximal, distal, and shaft hydatidosis.

Case. A 24-year-old patient derived from another center with a diagnosis of delayed healing of left humerus diaphyseal fracture (non-dominant side) with four months of evolution. He had been treated with a brace. At the moment of admission, he had pain and limited motion of his shoulder and elbow. Anteroposterior and lateral radiological images of the humerus showed a line of oblique fracture in the distal third of the diaphysis, and heterogeneous osteolytic and multiloculated images along the entire humerus. The MRI showed pathological images that compromised the entire diaphysis of the humerus and soft tissues at the expansive fracture site, with thinning of the cortical bones. It was decided to perform a biopsy, which resulted in hydatid cysts. The wound of the puncture evolved with the secretion of the hydatids prolonging its healing. Surgical treatment was planned in order to save the limb, given the aggressiveness and expansion of the lesions. Treatment. Oncological resection of the humerus and total replacement of the same with a non-conventional prosthesis designed for the patient was indicated. The surgery was performed using an anteromedial approach extended to the entire diaphysis, achieving total resection. The macroscopic anatomy showed involvement of the entire humerus, including the proximal and distal end with fistulas and vesicles with parasites. The prosthesis at its proximal end consisted of a unipolar head with provisions to attach abductors Fig. 2.

Soft tissue reconstruction was achieved by suturing the divided tendons, i.e., tendons of the rotator cuff, pectoralis major, subscapularis, latissimus dorsi, and teres major, and extensors and flexors in the elbow to the prosthesis. The arm was placed in a sling for six weeks, and the first ROM exercises are started at the elbow. Passive ROM exercises of the shoulder started at four weeks, followed by active exercises at three months and eight weeks after the operation. The patient was also treated with albendazole (15 mg/kg/ d orally) before the operation for one month and after the operation for six months as adjuvant therapy. Result: After two years of follow-up, the patient was pain-free with a range of elbow mobility of 15–90° of flexion-extension, with 0° of pronosupination, and wrist and hand with a full range of motion. At his shoulder he had 30° of anterior flexion, rot ext of 10° and internal rot at the gluteal level.

**Fig. 3:**
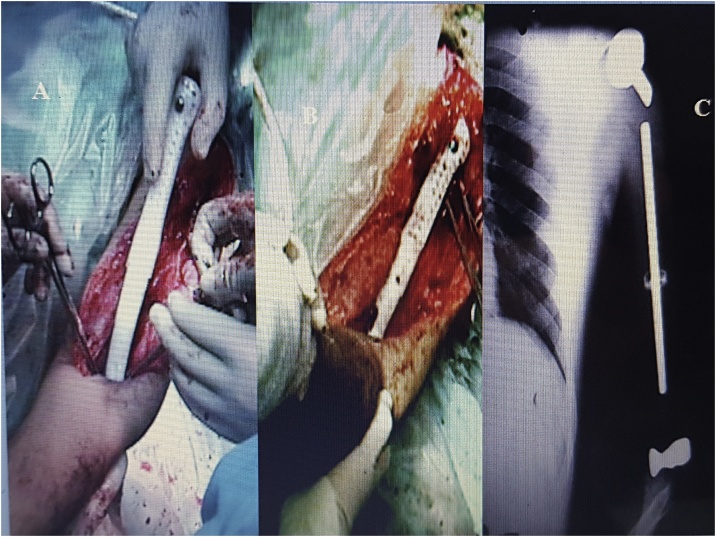
A, B total humerus prosthesis. C postoperative X-rays.

**Fig. 4:**
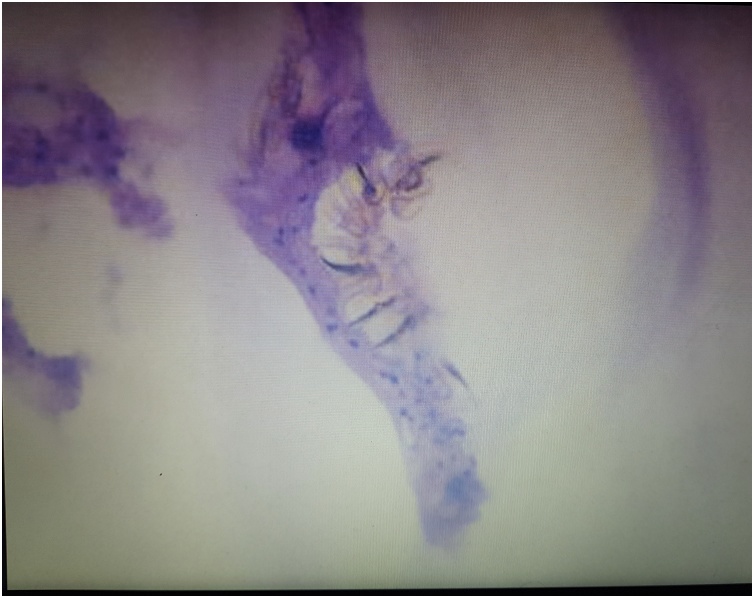
Histopatology showing hydatid cysts.

No local or general disease recurrences were observed. The patient could perform only administrative tasks. The ASES score was 58.33 and Mayo elbow was 65, Quick Dash was 42.3. At present, the patient does not perform tasks or effort with the affected upper limb but can use the hand and the elbow Fig. 3, Fig. 4.

## 2 Discussion

Hydatidosis is an endemic disease in different areas of the planet. The primary form in the bones is rare; the humerus has hardly been reported. There is scant information on severe cases with involvement of the entire humerus, including both epiphyses.

The information published following our literature review is case reports with local treatment (curettage), most of them without prolonged follow-up. Surgery is usually the first treatment option. There have been reports of local resections and osteosynthesis, but we believe that in this particular case is not feasible.

Curettage in the humerus has been proposed, but multiple local recurrences have been reported [10], as well as the need for multiple reoperations due to local recurrences and loosening of osteosynthesis in fractures. In addition, short follow-ups or lack thereof are reported [[11], [12], [13]].

The use of drugs, such as albendazole also seems to be controversial. In an epidemiological study of 17 patients diagnosed with hydatidosis, eight were found with bone involvement, two patients in the spine, 3 in the pelvis, and only 1 in the humerus and 1 in the tibia. None received albendazole, and no treatment or evolution was reported [14].

The only real curative procedure seems to be a radical excision. However, it was only possible in 16 percent of the cases [14]: when the axial bone-spine, the pelvic bone or the femur are affected.

In many cases, combined therapies based on surgery and antiparasitic drugs are used. However, it is known that the cure is difficult, and recurrence is frequent. We have not identified information on humerus hydatidosis that involves all the bone and its treatment with total resection of the humerus with an endoprosthesis.

The radical resection as salvage of the entire humerus is a challenge. The options are the allograft and the halo prostheses. For both procedures, functional expectations are scarce mainly at shoulder level, functioning as spacers, and to preserve the function of the wrist and hand. In addition to other general complications such as infections and loosening.

Regarding the total endoprostheses, they present functional complications in the shoulder and elbow. There is little experience with tumors, and they are considered a reasonable option in terms of elbow and hand preservation [15]. Another problem is usually the migration and dislocations of the prosthesis [16]. Natarajan et al· reported the use of a customized megaprosthesis for the reconstruction of bone defects secondary to hydatid bone resection in 3 patients, with excellent results and without recurrences. However, this study had a brief follow-up, which underestimates the likelihood of recurrent infection or loosening of the implant, which complicates prosthetic reconstruction [8].

Many authors have reported on the limited active ROM of the shoulder after the proximal humeral and THERs [17,18]. Careful soft tissue reconstruction with reattachment of the rotator cuff tendons did not seem to improve the results. Despite the poor movement at the shoulder, patients had excellent manual dexterity and functional use of their hands.

The use of tubes and vascular graft for the capsular reconstruction and for the reattachment of the soft tissue in addition to tendon transfers has not shown better results in terms of joint mobility [17,19].

In the present case, the functional result was not good at the level of the shoulder but functional in the wrist, hand, and elbow, and without recurrence of the disease until the last follow-up. It was planned with that objective.

Recently, techniques with better shoulder results have been reported [17]. These authors used a fully expandable humerus prosthesis in a girl with osteosarcoma.

We did not find reports about massive allograft reconstruction in the humerus.

Until the last follow-up of our patient, we did not find glenohumeral subluxation. Marulanda et al. observed no shoulder dislocations in their series of 16 patients with proximal humeral replacement reconstructed with an aortograft mesh. They found that the sleeve created by the aortograft allows for mechanical restraint and facilitates soft tissue reconstruction after tumor resection [20]. Puri and Gulia reported a similar experience with no observation of shoulder dislocations in their series of 20 patients with total humeral [21].

## 3 Conclusions

The cases of hydatidosis in the humerus are infrequent; the management is not standardized, the local resection plus graft and osteosynthesis conserving the bone has shown complications and recurrences. To aim for healing, a radical procedure is necessary with resections and reconstructions with prostheses. Although functional results may be limited, the preservation of the limbs and the function of the hand and wrist are feasible.

## Funding

We did not receive any funding.

## Ethical approval

The institutional Ethical Committee approved the retrospective medical chart review.

## Consent

Informed consent was obtained from the patient included in the study, and the institutional Ethical Committee approved the retrospective medical chart review.

## Author’s contribution

Juan Martin Patiño and Alejandro Jose Ramos Vertiz contributed to study design, data collection, data interpretation, and writing.

## Registration of research studies

This manuscript is not a human study, but a case report.

## Guarantor

Juan Martin Patiño.

## Provenance and peer review

Not commissioned, externally peer-reviewed.

## Declaration of Competing Interest

We do not have conflicts of interest to declare.
